# Genome-wide association analysis for maize stem Cell Wall-bound Hydroxycinnamates

**DOI:** 10.1186/s12870-019-2135-x

**Published:** 2019-11-27

**Authors:** A. López-Malvar, A. Butrón, L. F. Samayoa, D. J. Figueroa-Garrido, R. A. Malvar, R. Santiago

**Affiliations:** 1 Facultad de Biología, Departamento de Biología Vegetal y Ciencias del Suelo, Universidad de Vigo, As Lagoas Marcosende, 36310 Vigo, Spain; 2 Agrobiología Ambiental, Calidad de Suelos y Plantas (UVIGO), Unidad Asociada a la MBG (CSIC), Vigo, Spain; 3 Misión Biológica de Galicia (CSIC), Pazo de Salcedo, Carballeira 8, 36143 Pontevedra, Spain; 4 Department of Crop and Soil Sciences, North Carolina State University Raleigh, Raleigh, NC 27695-7620 USA

**Keywords:** Association mapping, Hydroxycinnamic acid, Diversity panel, Cell-wall fortification, *Zea mays*

## Abstract

**Background:**

The structural reinforcement of cell walls by hydroxycinnamates has a significant role in defense against pests and pathogens, but it also interferes with forage digestibility and biofuel production. Elucidation of maize genetic variations that contribute to variation for stem hydroxycinnamate content could simplify breeding for cell wall strengthening by using markers linked to the most favorable genetic variants in marker-assisted selection or genomic selection approaches​.

**Results:**

A genome-wide association study was conducted using a subset of 282 inbred lines from a maize diversity panel to identify single nucleotide polymorphisms (SNPs) associated with stem cell wall hydroxycinnamate content. A total of 5, 8, and 2 SNPs were identified as significantly associated to *p*-coumarate, ferulate, and total diferulate concentrations, respectively in the maize pith. Attending to particular diferulate isomers, 3, 6, 1 and 2 SNPs were related to 8–*O*–4 diferulate, 5–5 diferulate, 8–5 diferulate and 8–5 linear diferulate contents, respectively. This study has the advantage of being done with direct biochemical determinations instead of using estimates based on Near-infrared spectroscopy (NIRS) predictions. In addition, novel genomic regions involved in hydroxycinnamate content were found, such as those in bins 1.06 (for FA), 4.01 (for PCA and FA), 5.04 (for FA), 8.05 (for PCA), and 10.03 and 3.06 (for DFAT and some dimers).

**Conclusions:**

The effect of individual SNPs significantly associated with stem hydroxycinnamate content was low, explaining a low percentage of total phenotypic variability (7 to 10%). Nevertheless, we spotlighted new genomic regions associated with the accumulation of cell-wall-bound hydroxycinnamic acids in the maize stem, and genes involved in cell wall modulation in response to biotic and abiotic stresses have been proposed as candidate genes for those quantitative trait loci (QTL). In addition, we cannot rule out that uncharacterized genes linked to significant SNPs could be implicated in dimer formation and arobinoxylan feruloylation because genes involved in those processes have been poorly characterized. Overall, genomic selection considering markers distributed throughout the whole genome seems to be a more appropriate breeding strategy than marker-assisted selection focused in markers linked to QTL.

## Background

In terms of agricultural land use and production, maize (*Zea mays* L.) is one of the most important crops worldwide. In addition to the general uses of maize grain as food, feed, or raw material for generating industrial derivatives, maize stover could be a profitable byproduct for ethanol production, whereas the whole plants can also be used to produce silage for feeding cattle.

Accessibility, extensibility, and digestibility of maize stem tissues may determine important characteristics of the maize stem such as maize resistance to stem borer pests and stem diseases, feedstuff quality and degradability and suitability for ethanol production [1]. These characteristics depend greatly on cell wall functionality and structure, which are controlled by the composition and organization of individual cell wall components [1]. Maize cell walls are mainly composed of cellulose embedded in a hemicellulose matrix and lignin. Among cell wall components, cell wall bound hydroxycinnamates, which are derived from the phenylpropanoid pathway and originate from tyrosine and phenylalanine, play a key role in cell wall structural reinforcement. The main hydroxycinnamates in the maize stem are esters of ferulic acid (FA) and *p*-coumaric acid (PCA), which both contribute to cell wall stiffening and fortification [2]. PCA acts as a radical transfer agent to promote the formation of sinapyl alcohol and lignin radicals being esterified to the Y-position of the side chains of S lignin units [3, 4]. On the other hand, FA could be found within the cell wall esterified to arabinosyl residues of arabinoxylan chains which are thereafter cross-linked via ether bonds to G units of lignins [5, 6]. In addition, it is important to note that FA forms dimers via peroxidase- or laccase-mediated oxidative coupling, and the resulting diferulates (DFAs) cross-links hemicellulose chains (7).

In pest resistance several studies, focused on the study of differences for cell wall components among contrasting materials for resistance to stem borers, have suggested that cell wall strengthening could be a constitutive resistance mechanism to protect maize plants against the attack [7, 8]. The effects of cell wall-bound hydroxycinnamate content on stem borer resistance have been thoroughly investigated, identifying higher concentrations of hydroxycinnamates in resistant inbred lines [9, 10]. In forage species fiber comprises 300–800 μg/g dry matter, and represents the greatest source of energy for ruminants. Unfortunately, less than 50% of this fiber content is actually digested and used by the animal, mainly because of the recalcitrant role of specific cell wall components. The variability in the digestibility of the maize cell wall, which is often greater than 50%, is due, in part, to variability for lignin content, but also to differences for lignin composition itself and cell wall cross-linkage mediated by ferulates and DFAs [11]. For instance, Jung et al. (2011) observed that dry matter intake, and milk production were greater in cows fed with a diet containing W23*sfe*, a maize mutant with decreased ferulate content, than in cows fed with forage from the wild-type W23. In the same study in vivo digestibility of cell walls was greater on W23*sfe* [12].

In ethanol production, the two key parts of the maize plant that can be converted to bioethanol are the grain, which is mainly made up of starch, and the debris (stem and leaves), which predominantly contains lignin and cellulosic components. Ethanol produced from non-grain plant material is defined as cellulosic or lignocellulosic ethanol. Lignocellulose in maize is composed of 33.1% hemicellulose, 39.4% cellulose, and 14.9% lignin [13, 14]. The degradation of carbohydrates to the constituent sugar monomers (saccharification) provides the substrates for the fermentation required for yeast-mediated ethanol production. However, the largest cell wall component, cellulose, is not particularly susceptible to deconstruction and is closely interconnected with hemicelluloses and lignin. Cross-linking of lignin to arabinoxylan by hydroxycinnamates makes cell walls highly recalcitrant to biomass degradation [15, 16].

Overall, the mechanical resistance due to higher hydroxycinnamate content and lignification makes maize tissues more recalcitrant to damage by insects, less digestible for ruminants, and less suitable for biofuel production [17–19]. The study of the maize functional genetic variability for each hydroxycinnamate component could be crucial to identify relevant genetic variants that may be incorporated into selection programs to breed maize varieties for multiple uses.

Some maize genomic regions mediating hydroxycinnamate accumulation have been detected throughout the genome [17, 20, 21]. Most of these previous studies involved biparental populations and were useful for detecting large regions of interest by analyzing those populations and relatively few markers. Genome-Wide Association Studies (GWAS) enable high-resolution mapping of QTL to narrow genomic regions where the searching for genes contributing to trait variability is feasible. This technique has been extensively used to identify maize genetic variants significantly associated with yield and agronomic traits [22], resistance to biotic and abiotic stresses [23], specific cell wall components such as lignin, cellulose, hemicellulose, detergent fibers, and in vitro digestibility of dry matter [24]. This study constitutes the first high resolution association mapping analysis made to detect QTLs associated to hydroxycinnamate content and has been performed using a diversity panel that contents most genetic variability of maize adapted to temperate areas. Additionally, materials were phenotyped based on wet chemistry determinations, which are more precise than estimations based on Near-Infrared Spectroscopy or Fourier-Transform Infrared Spectroscopy predictions. The objective of this study was to identify genomic regions that could make relevant contributions to the genetic variability for cell wall-bound hydroxycinnamate content in a diverse inbred panel and to propose candidate genes for those regions.

## Results

A subset of 270 inbred lines from this diversity panel was evaluated for cell wall-bound hydroxycinnamates. We quantified by liquid chromatography the two main cell wall hydroxcinnamic acids, *p*-coumaric and ferulic acid, and four ferulic acid dimers (DFA 5–5, DFA 8–*O*–4, DFA 8–5b, and DFA 8–5 l). As DFA 8–5 l and 8–5b are different isoforms of DFA 8–5, we calculated DFA 8–5 as the sum of both isoforms. Besides, the sum of total dimers (DFAT), total monomers (MONOTOT) and total hydroxycinnamates (FENTOT) were also calculated.

### Means, analysis of variance, and Heritabilities

Significant differences were detected among inbred lines for all cell wall-bound hydroxycinnamates (see Additional file 1). Significant genotype-by-environment interactions were observed for individual dimers and DFAT (Data not shown). High heritability values (> 0.70) were estimated for every trait (Table 1).

**Table 1:** Heritability estimates for the traits under study

Trait	***h***^**2**^
PCA	0.879 ± 0.015
FA	0.825 ± 0.022
DFA 5–5	0.792 ± 0.026
DFA 8–*O*–4	0.811 ± 0.024
DFA 8–5 l	0.787 ± 0.023
DFA 8–5b	0.734 ± 0.033
DFA 8–5	0.768 ± 0.029
DFAT	0.797 ± 0.025
MONOTOT	0.871 ± 0.016
FENTOT	0.868 ± 0.016

PCA: *p*-coumaric acid; FA: ferulic acid; DFA 8–5 l: 8–5 linear diferulic acid; DFA 5–5: 5–5 diferulic acid; DFA 8–*O*–4: 8–*O*–4 diferulic acid; DFA 8–5b: 8–5 benzofuran diferulic acid; DFA 8–5: sum (DFA 8–5 l + DFA 8–5b); DFAT: total diferulates sum (DFA 5–5 + DFA 8–*O*–4 + DFA 8–5b + DFA 8–5 l); MONOTOT: total monomers (PCA + FA); FENTOT: total hydroxycinnamates (PCA + FA + DFAT)

### Correlation analysis

All correlation (phenotypic and genotypic) coefficients between traits were positive and significant except for the correlation coefficient between DFA 8–5 l and PCA and between DFA 8–5 l and FA. Additionally, PCA was strongly correlated with the total monomer content (MONOTOT) (r_g_ = r_p_ = 0.98) and the total cell wall-bound phenolic acid content (FENTOT) (r_g_ = r_p_ = 0.98). Both correlation coefficients between FA and dimers, as well as the correlation coefficients among the diverse dimers were high and significant (r > 0.70) (Table 2). The strong correlations suggested that MONOTOT and FENTOT are mainly determined by the PCA concentration. Thus, MONOTOT and FENTOT are not further addressed.

**Table 2:** Genotypic^1^ (above diagonal) and phenotypic^2^ (below diagonal) correlation coefficient estimates for each pair of traits

	PCA	FA	DFA 5–5	DFA 8–*O*–4	DFA 8–5	DFA 8–5b	DFA 8–5 l	DFAT	MONOTOT	FENTOT
PCA		0.47*	0.41*	0.35*	0.44*	0.47*	NS	0.42*	0.98*	0.98*
FA	0.47*		0.84*	0.80*	0.74*	0.72*	NS	0.80*	0.63*	0.65*
DFA5–5	0.43*	0.81*		0.96*	0.96*	0.96*	0.89*	0.99*	0.55*	0.57*
DFA 8–*O*–4	0.39*	0.79*	0.93*		0.94*	0.93*	0.91*	0.98*	0.48*	0.50*
DFA 8–5	0.47*	0.74*	0.91*	0.92*		1.00	0.96*	0.99*	0.55*	0.57*
DFA 8–5b	0.46*	0.71*	0.89*	0.90*	1.00		0.91*	0.99*	0.57*	0.59*
DFA 8–5 l	NS	NS	0.95*	0.95*	1.00	0.99*		0.99*	0.54*	0.57*
DFAT	0.45*	0.79*	0.96*	0.97*	0.98*	0.96*	0.96*		0.53*	0.55*
MONOTOT	0.98*	0.64*	0.56*	0.52*	0.58*	0.56*	0.57*	0.57*		1.00
FENTOT	0.98*	0.66*	0.58*	0.54*	0.60*	0.58*	0.60*	0.59*	1.00	

PCA: *p*-coumaric acid; FA: ferulic acid; DFA 8–*O*–4: 8–*O*–4 diferulic acid; DFA 5–5: 5–5 diferulic acid; DFA 8–5: sum (DFA 8–5 l + DFA 8–5b); DFA 8–5b: 8–5 benzofuran diferulic acid; DFA 8–5 l: 8–5 linear diferulic acid; DFAT: total diferulates sum (DFA 5–5 + DFA 8–*O*–4 + DFA 8–5b + DFA 8–5 l); MONOTOT: total monomers (PCA + FA); FENTOT: total hydroxycinnamates (PCA + FA + DFAT)

1: * Significant genotypic correlation coefficient because it exceeded twice its standard error; NS: Non-Significant

2: * Significant phenotypic correlation coefficient because it exceeded twice its standard error; NS: Non-Significant

### Association analysis

A marker was considered to be significantly associated with a trait at RMIP (Resample Model Inclusion Probability) values more than 0.50. We considered a +/− 150 kbp region around the significant SNP. Two SNPs were assigned to the same QTL when their confidence intervals overlapped. Consequently, 27 SNPs, which corresponded to 22 QTLs, were identified as significantly associated with cell wall-bound hydroxycinnamates, 5 SNPs were associated with PCA, 8 SNPs with FA, 3 SNPs with DFA 8–*O*–4, 6 SNPs with DFA 5–5, 1 SNP with DFA 8–5, 2 SNPs with DFA 8–5 linear and 2 SNPs with DFAT (Table 3). Among these associations, novel genomic regions involved in hydroxycinnamate content were found in bins 1.06 (for FA), 4.01 (for PCA and FA), 5.04 (for FA), 8.05 (for PCA), and 10.03 and 3.06 (for DFAT and some dimers). Minor and major frequency alleles contributed almost equally to increased PCA levels, while minor frequency alleles generally increased cell wall-bound FA and DFA concentrations. Differences between homozygous for minor and major frequency alleles at each significant SNP varied from 569 to 851 μg/g DW for PCA concentration, from 177 to 321 μg/g DW for FA concentration and from 55 to 56 μg/g DW for DFAT concentration. Differences for dimer concentrations were as follows: 15 to 17 μg/g DW for DFA 8–*O*–4, 6 to 15 μg/g DW for DFA 5–5, 12 μg/g DW for DFA 8–5 and 5 to 8 μg/g DW for DFA 8–5 l (Table 3). The percentages of variances explained by each significant SNP ranged from 7 to 10%. The significant SNPs found in the current study were distributed in bins 1.05, 1.06, 1.07, 1.11, 3.04, 3.06, 4.01, 5.04, 5.04, 8.05 and 10.03.

**Table 3:** SNP identification (SNP ID), additive effect and allelic variants for the SNP, proportion of total variance explained by the SNPs significantly associated with cell wall traits (PCA, FA, DFAT, DFA 8–*O*–4, DFA 8–5 l, DFA 8–5, DFA 5–5), and significance values for the association between the SNP and the phenotype (*P*-value and RMIP)

Trait^a^	QTL^b^	Marker^c^	Chr^d^	Bin^e^	Alleles^f^	(No)^g^	Add Effect^h^	P-value	RMIP^i^	R^2j^
PCA	qPCA_1_1	S1_174637686	1	1.05	C/T	108/147	568.75	4.3E-08	0.69	0.08
PCA	qPCA_1_2	S1_288696782	1	1.11	T/G	190/68	813.84	1.9E-07	0.85	0.09
PCA	qPCA_1_3	S1_108071292	1	1.05	T/G	147/100	851.22	6.5E-08	0.92	0.11
PCA	qPCA_1_3	S1_108071293	1	1.05	A/T	147/101	851.22	6.5E-08	0.92	0.11
PCA	qPCA_3_1	S3_20426421	3	3.04	C/G	79/170	697.11	6.9E-07	0.57	0.08
FA	qFA_1_1	S1_187590405	1	1.06	T/C	125/133	177.91	1.9E-06	0.52	0.08
FA	qFA_1_2	S1_220067811	1	1.07	C/T	23/239	304.04	1.6E-06	0.61	0.07
FA	qFA_1_2	S1_220067812	1	1.07	C/T	20/237	320.80	1.2E-06	0.54	0.08
FA	qFA_1_3	S1_295476338	1	1.11	T/C	35/222	269.14	1.5E-08	0.81	0.08
FA	qFA_1_3	S1_295476576	1	1.11	C/T	37/223	243.99	6.4E-08	0.66	0.09
FA	qFA_4_1	ss4_10220935	4	4.01	C/A	170/83	181.03	1.9E-07	0.66	0.08
FA	qFA_5_1	ss5_169927760	5	5.04	A/G	81/182	185.18	1.1E-06	0.51	0.08
FA	qFA_8_1	S8_138322127	8	8.05	T/C	22/240	280.72	3.2E-07	0.57	0.08
DFAT	qDFAT_10_1	S10_22521088	10	10.03	A/G	15/240	55.93	7.9E-07	0.51	0.07
DFAT	qDFAT_3_1	S3_184608458	3	3.06	C/T	15/245	55.34	8.4E-08	0.5	0.08
DFA 8–*O*–4	qDFA8o4_1_1	S1_220067811	1	1.07	C/T	23/239	15.07	8.1E-07	0.69	0.08
DFA 8–*O*–4	qDFA8o4_1_1	S1_220067812	1	1.07	C/T	20/237	15.49	4.7E-07	0.54	0.08
DFA 8–*O*–4	qDFA8o4_10_1	S10_22521088	10	10.03	A/G	15/240	16.70	3.2E-07	0.51	0.07
DFA 5–5	qDFA55_1_1	S1_297490295	1	1.11	A/G	99/161	5.81	2.0E-06	0.57	0.07
DFA 5–5	qDFA55_1_2	S1_220067811	1	1.07	C/T	23/239	10.78	3.1E-07	0.62	0.08
DFA 5–5	qDFA55_1_2	S1_220067812	1	1.07	C/T	20/237	11.94	9.1E-08	0.7	0.08
DFA 5–5	qDFA55_3_1	ss3_60941077	3	3.04	G/A	38/229	8.39	9.8E-07	0.52	0.07
DFA 5–5	qDFA55_3_2	S3_184608458	3	3.06	C/T	15/245	14.73	6.49E-08	0.77	0.10
DFA 5–5	qDFA55_10_1	S10_22521088	10	10.03	A/G	15/240	13.24	3.9E-07	0.75	0.08
DFA 8–5	qDFA85_1_1	S1_297490295	1	1.11	A/G	99/161	12.04	9.7E-07	0.51	0.07
DFA 8–5 l	qDFA85l_1_2	S1_297490295	1	1.11	A/G	99/161	4.69	3.9E-07	0.64	0.08
DFA 8–5 l	qDFA85l_1_1	S1_220067811	1	1.07	C/T	23/239	8.30	4.1E-07	0.56	0.08

a: PCA: *p*-coumaric acid; FA: ferulic acid; DFA 8–5 l: 8–5 linear diferulic acid; DFA 5–5: 5–5 diferulic acid; DFA 8–*O*–4: 8–*O*–4 diferulic acid; DFA 8–5b: 8–5 benzofuran diferulic acid; DFA 8–5: sum (DFA 8–5 l + DFA 8–5b); DFAT: total diferulates sum (DFA 5–5 + DFA 8–*O*–4 + DFA 8–5b + DFA 8–5 l)

b: The number before the underscores indicates the chromosome and the number after the underscores indicates the QTL within the chromosome

c: The number before the underscores indicates the chromosome number and the number after the underscore indicates the physical position in bp within the chromosome

d: Chromosome

e: A bin is the interval that includes all loci from the leftmost or top Core Marker to the next Core Marker. The genetic maps are divided into 100 segments of approximately 20 centiMorgans designated with the chromosome number followed by a two-digit decimal [25]

f: The letter before the diagonal is the nucleotide with the larger value; and the letter after the diagonal is the nucleotide with the smaller value

g: No = Number of inbred lines homozygous for a determined allelic variant, The number before the diagonal represents the number of homozygous with the largest mean value; and the number after the diagonal the number of homozygous with the smaller mean value

h: Additive effect (μg/g DW): the additive effect was calculated as half the difference between the mean of the homozygous for the allele with the largest value and the mean of the homozygous for the allele with the smallest value

i: RMIP: resample model inclusion probability

j: Phenotypic variance explained by each marker

### Candidate gene selection

The genes containing or physically close to SNPs significantly (RMIP > 0.5) associated with traits were identified and characterized according to the maize B73 reference genome assembly (version 4). Analyses of +/− 150kbp regions surrounding significant SNPs resulted in the identification of 111 genes listed in Table 4. Sixty-three of these genes have not been so far characterized.

**Table 4:** Complete list of candidate genes for hydroxycinnamates^a^ identified in a maize diversity panel. Genes in bold are the ones mentioned in the manuscript

Trait^a^	Marker	Chr^b^	Bin^c^	Physical position^d^	Gene ^e^	Gene Function^f^
PCA	S1_174637686	1	1.05	174,637,686	Zm00001d031086	Nced2 - nine-cis-epoxycarotenoid dioxygenase2
					Zm00001d031088	Putative kelch repeat-containing protein containing ser/thr protein kinase family protein
					**Zm00001d031090**	GTP binding protein
					Zm00001d031091	Hypothetical protein
PCA	S1_108071292	1	1.05	108,071,292	Zm00001d030173	Calmodulin-binding receptor-like cytoplasmic kinase 3
PCA	S1_108071293	1	1.05	108,071,293	Zm00001d030174	Lecithin-cholesterol acyltransferase-like 1
					Zm00001d030176	Hypothetical protein
					Zm00001d030177	Hypothetical protein
FA	S1_187590405	1	1.06	187,590,405	Zm00001d031426	Serine/threonine-protein kinase UCNL
					Zm00001d031427	Leucine-rich repeat receptor-like protein kinase PEPR1
					**Zm00001d031428**	Monogalactosyldiacylglycerol synthase 2 chloroplastic
					Zm00001d031429	pfkB-like carbohydrate kinase family protein
					Zm00001d031430	Hypothetical protein
FA DFA 8–*O*–4 DFA 5–5	S1_220067812	1	1.07	220,067,812	Zm00001d032339	Hypothetical protein
					Zm00001d032342	Leucine-rich repeat receptor protein kinase EMS1
					Zm00001d032343	sdg103 - SET domain group 103)
FA DFA 8–*O*–4 DFA 8–5 l DFA 5–5	S1_220067811	1	1.07	220,067,811	Zm00001d032344	Receptor-like protein kinase HSL1
					Zm00001d032345	Nudix hydrolase 15 mitochondrial
					**Zm00001d032346**	UDP-glucose 4-epimerase 4
					Zm00001d032347	DNAJ heat shock family protein
					Zm00001d032348	Zinc finger BED domain-containing protein RICESLEEPER 2
					Zm00001d032350	Hypothetical protein
PCA	S1_288696782	1	1.11	288,696,782	Zm00001d034469	PRA1 prenylated Rab receptor 2
					Zm00001d034471	Ketol-acid reductoisomerase chloroplastic
					Zm00001d034473	Metal transporter Nramp6-heavy metal uptake
					Zm00001d034475	WRKY12 -WRKY-transcription factor 12
					Zm00001d034476	Hypothetical protein
					Zm00001d034477	Hypothetical protein
					Zm00001d034479	hon110 histone one H1
					Zm00001d034480	Seed Specific protein Bn15D14A
					Zm00001d034482	Hypothetical protein
					Zm00001d034483	Hypothetical protein
					Zm00001d034484	Hypothetical protein
					Zm00001d034485	rtl4- reversion-to-ethylene sensitivity1 like4
					**Zm00001d034486**	GTP binding protein
FA	S1_295476576	1	1.11	295,476,576	**Zm00001d034727**	Polygalacturonase
					Zm00001d034728	Uncharacterized
					Zm00001d034729	Carbohydrate-binding X8 domain superfamily protein
					Zm00001d034730	sbp17 - SBP-transcription factor 17
FA	S1_295476338	1	1.11	295,476,338	Zm00001d034731	Short-chain dehydrogenase reductase 3b
					**Zm00001d034732**	gpat3 - glycerol-3-phosphate acyltransferase3
					Zm00001d034733	Galactokinase
					Zm00001d034734	Nucleic acid-binding proteins superfamily
					Zm00001d034735	Methyltransferase superfamily protein
					Zm00001d034736	cps1 - chloroplast protein synthesis1
					Zm00001d034737	Hypothetical protein
					Zm00001d034739	Fumarylacetoacetate FAA hydrolase family
					**Zm00001d034740**	Epoxide hydrolase 2
					Zm00001d034741	Hypothetical protein
					Zm00001d034742	Hypothetical protein
					Zm00001d034743	OsNAC protein-like
					Zm00001d034744	Pentatricopeptide repeat-containing protein
DFA 8–5 l DFA 8–5 DFA 5–5	S1_297490295	1	1.11	297,490,295	Zm00001d034817	Protein phosphatase 2A regulatory subunit B′
					Zm00001d034818	GDSL esterase/lipase
					Zm00001d034819	Putative replication protein
					Zm00001d034820	Defective in cullin neddylation protein
					Zm00001d034822	Hypothetical protein
					Zm00001d034823	ALBINO3-like protein 1 chloroplastic
					Zm00001d034824	aldose-1-epimerase
					Zm00001d034826	Elongation factor G-1 mitochondrial
PCA	S3_20426421	3	3.04	20,426,421	**Zm00001d039926**	WAK1 - OsWAK receptor-like cytoplasmic kinase OsWAK-RLCK
					Zm00001d039927	Hypothetical protein
					Zm00001d039928	Hypothetical protein
					Zm00001d039929	Hypothetical protein
					Zm00001d039930	Hypothetical protein
					Zm00001d039931	Leaf rust 10 disease-resistance locus receptor-like protein kinase-like 1.1
					Zm00001d039932	Hypothetical protein
					Zm00001d039933	16.9 kDa class I heat shock protein 1
					Zm00001d039935	hsp17.2 - heat shock protein17.2
					Zm00001d039936	hsp12 - heat shock protein12
					Zm00001d039937	TIM-barrel signal transduction protein isoform 2
					Zm00001d039938	Calmodulin-binding transcription activator 1
DFAT DFA 5–5	S3_184608458	3	3.06	184,608,458	Zm00001d043050	RING-H2 finger protein ATL74
					Zm00001d043052	Unknown Endoplasmic reticulum vesicle transporter protein
					Zm00001d043056	Serine/threonine-protein kinase NAK
					Zm00001d043057	Unknown Protein kinase
					Zm00001d043058	Leucine-rich repeat receptor-like serine/threonine-protein kinase IRK
DFA 5–5	ss3_60941077	3	3.04	60,941,077	Zm00001d040735	invan10 - invertase alkaline neutral10
					**Zm00001d040737**	ga2ox5 - gibberellin 2-oxidase5
FA	ss4_10220935	4	4.01	10,220,935	Zm00001d048968	Leucine-rich repeat receptor-like serine/threonine-protein kinase BAM1
					Zm00001d048969	phd28 - PHD-transcription factor 28
					**Zm00001d048970**	Glycerophosphodiester phosphodiesterase GDPD4
					Zm00001d048972	Hypothetical protein
					Zm00001d048973	Hypothetical protein
					Zm00001d048974	Glycine cleavage system H protein 2 mitochondrial
					Zm00001d048975	Hypothetical protein
					Zm00001d048977	Hypothetical protein
					Zm00001d048978	F-box domain containing protein
					Zm00001d048979	Probable sucrose-phosphate synthase 5
FA	ss5_169927760	5	5.04	169,927,760	**Zm00001d016731**	Digalactosyldiacylglycerol synthase 1
					Zm00001d016732	bzip103 - bZIP-transcription factor 103
					Zm00001d016733	NDR1/HIN1-like 1
					Zm00001d016734	Hypothetical protein
					Zm00001d016735	Protein SMAX1-LIKE 3
					Zm00001d016736	2-cys peroxiredoxin BAS1
					Zm00001d016737	e2f4 - E2F-DP-transcription factor 24)
					**Zm00001d016738**	Pectinesterase inhibitor domain containing protein
					Zm00001d016740	Hypothetical protein
					**Zm00001d016741**	Pectinesterase inhibitor domain containing protein
					Zm00001d016742	Hypothetical protein
					Zm00001d016743	Hypothetical protein
					Zm00001d016744	Hypothetical protein
FA	S8_138322127	8	8.05	138,322,127	Zm00001d011187	bhlh92 - bHLH-transcription factor 92
					Zm00001d011188	3′-5′ exonuclease domain-containing protein / K homology domain-containing protein / KH domain-containing protein
					Zm00001d011189	DUF3511 domain protein
					Zm00001d011190	Hypothetical protein
					Zm00001d011192	Carbamoyl-phosphate synthase large chain, chloroplastic
					Zm00001d011193	Integral membrane protein
					Zm00001d011195	Putative protein phosphatase 2C 74
DFAT DFA 8–*O*–4, DFA 5–5	S10_22521088	10	10.03	22,521,088	**Zm00001d023813**	Polygalacturonase, pectin
					Zm00001d023815	Triacylglycerol lipase

a: a: PCA: *p*-coumaric acid; FA: ferulic acid; DFA 8–5 l: 8–5 linear diferulic acid; DFA 5–5: 5–5 diferulic acid; DFA 8–*O*–4: 8–*O*–4 diferulic acid; DFA 8–5b: 8–5 benzofuran diferulic acid; DFA 8–5: sum (DFA 8–5 l + DFA 8–5b); DFAT: total diferulates sum (DFA 5–5 + DFA 8–*O*–4 + DFA 8–5b + DFA 8–5 l)

b: Chr: Chromosome

c: A bin is the interval that includes all loci from the leftmost or top Core Marker to the next Core Marker. The genetic maps are divided into 100 segments of approximately 20 centiMorgans designated with the chromosome number followed by a two-digit decimal [26]

d: Physical position of the marker in the B73 Reference Genome version 2

e: Name of the gene in B73 Reference Genome version 4

f: Gene function according to Zm-B73 reference form Gramene

## Discussion

### Heritabilities and correlations

The high heritabilities observed in the current study are consistent with the results of previous studies [17, 21]. Our data indicate that additive effects are more important than additive × environment interaction effects in the inheritance of hydroxycinnamates in the pith of maize stems, and, consequently, high responses to selection, using any of these traits as selection criteria, would be expected [27]. Based on the evidenced correlation between greater strengthening of the cell wall and the increased hydroxycinnamate content, these traits could be used as indirect selection criteria for the improvement of more complex traits such as pest resistance or forage digestibility [28].

The correlations between traits follow as well the trends reported in the literature [29, 30]. FA, particular dimers and DFAT show co-variation, meanwhile co-variations of PCA and any other hydroxycinnamate compounds were not relevant: Therefore, most genetic variability detected would not be consequence of variations for genes of the common metabolic pathway of these hydroxycinnamates.

### QTL co-localization

Significant SNPs were usually located in bins where QTL for hydroxycinnamates (PCA, FA, total diferulates and dimers) were found in previous studies (detailed below). However, novel genomic regions involved in hydroxycinnamate content were also found, such as those in bins 1.06 (for FA), 4.01 (for PCA and FA), 5.04 (for FA), 8.05 (for PCA), and 10.03 and 3.06 (for DFAT and some dimers).

SNPs significantly associated to FA content were found within the supporting intervals of QTLs detected for the same trait by Barriére et al. [29, 31], whereas markers associated with PCA content were included within QTLs for PCA detected by Santiago et al. [21], Barrière et al. [17, 29, 31], and Courtial et al. [32]. Similarly, significant SNPs for DFAT and individual dimers co-localized with QTLs previously published for those traits [31, 33, 34]. It is important to note that previously detected QTLs were mostly identified in bi-parental populations with lower level of resolution. Regarding the correlation coefficients among different traits, we would expect extensive co-localizations between QTLs for FA and individual and total dimers because the genotypic correlation coefficients among those traits were high. However, we only found one genomic region at bin 1.07 where QTLs for FA, DFA 8–*O*–4, DFA 8–5 l and DFA 5–5 co-localized. This could be explained by the high percentage the unexplained phenotypic variability, factors such as low frequency of minor alleles, small additive effects of genes and/or low density of markers could be responsible.

In the same regions, QTLs for resistance to pests [23, 35–37], animal degradability [24, 29, 38] and fuel production [29, 31] have already been reported (See Adittional File 2), supporting that the variation for cell wall bound hydroxycinnamates could have a significant role on these three aspects. Marker-assisted breeding programs to indirectly improve maize resistance to insect attack could focus on genomic regions where QTLs for hydroxycinnamates across different populations are found, as well as QTLs for increased resistance to stem borers; especially, if associated unfavorable effects for biofuel production and/or animal digestibility are not found as in bins 1.06 and 3.06. However, the lack of significant co-localizations does not mean that linked and/or pleiotropic genes do not exist in that region because detection power in the present study would not be enough to uncover all genomic regions involved in any trait. Therefore, we propose to implement genomic selection for increased cell-wall hydroxycinnamate content in a population with a high genetic diversity in order to better establish indirect effects of selection for increased cell-wall strength on resistance to stem borers, animal digestibility and/or biofuel production. In addition, effects on other cell-wall components such as lignin content or composition that also have impact on cell-wall strengthening would be desirable.

### Candidate genes

Key genes implicated in monolignols and *p*-hydroxycinnamic acid biosynthesis are known, and none of them were located at distances shorter than +/− 0.15 Mbp from SNPs significantly associated to hydroxycinnamates. Even though, little is known about the genes implicated in dimerization by peroxidase-mediated oxidative coupling of FA residues to form ferulic acid oligomers [39, 40]. Similarly, genes involved in feruloylation of arabonoxylans (AX) are poorly described; there are some hypothesis that propose CoA-acyl transferases in the PF02458 family as good candidates for feruloylation as the modification of CoA-acyl transferase genes changes the level of esterified ferulates and diferulates in the cell wall, although their roles are inconclusive [41, 42]. Recent studies describe that suppression of a single BAHD gene in *Setaria viridis* causes large, stable decreases in cell wall feruloylation and increases biomass digestibility [43]. However, we cannot rule out that some of the uncharacterized candidate genes and hypothetical proteins noted in the current study could be genes involved in the dimerization, specifically those located in bins 3.06 and 10.03, where significant SNPs for dimers have been found but not for FA, while uncharacterized candidate genes in bins 1.07 and 1.11 that contain significant SNPs for FA and dimer contents could be implicated in feruloylation of arabinoxylans.

Among the genes found in the confidence interval of QTL significantly associated with PCA, we highlight three of them: two possible 4-coumarate ligases (Zm00001d031090, Zm00001d034486) and a cell wall kinase (Zm00001d039926). The 4-coumarate ligase is a key enzyme involved in the general phenylpropanoid metabolism, that catalyzes the conversion of 4-coumaric acid into 4-coumaroyl-CoA leading to the formation of precursors that serve as structural components for the biosynthesis of cell wall associated phenolics [44].

Cell wall kinases (WAKs) are members of the RLCKs (Receptor-like cytoplasmic kinase) super family which are one of the main candidates for sensing and controlling cell wall integrity [45, 46]. Some WAKs display carbohydrate binding activity interacting with the cell wall probably via pectin binding, which appears to influence a WAK-dependent signaling pathway regulating cell expansion and also involved in signaling during stress response and pathogen infection resistance. A WAK gene is proposed as the best candidate gene for the QTL for PCA at bin 3.04 although the specific function of this gene needs to be tested.

Genes annotated as probably involved in glycerolipid metabolism within the supporting intervals of four QTLs for FA were also found: a glycerol-3-phosphate acyltransferase 1 (Zm00001d034732), an Epoxide hydrolase (Zm00001d034740), a Monogalactosyldiacylglycerol synthase 2 (Zm00007a00030071), a glycerophosphodiester phosphodiesterase (Zm00001d048970) and a digalactosyldiacylglycerol synthase 1 (Zm00001d016731). These enzymes could be also be involved in biosynthesis of steryl glycosides and acylated steryl glycosides [47]. Increased acylated steryl glycosides has been associated to matured fiber cells compared to elongating fiber cells [48]. Total FA content was highest or increased during cell elongation and was lower or decreased thereafter [48, 49], likely reflecting the assembly of the expanding cell membranes during elongation and the shift to membrane maintenance (and increase in secondary cell wall content) in maturing fibers [48]. Moreover, for the QTL for FA, DFA 8–*O*–4, DFA 8–5 l and DFA 5–5 in bin 1.07, we propose an UDP-glucose 4-epimerase 4 (UGE4) gene (Zm00001d032346) as probable candidate gene. In Arabidopsis, UGE4 participates in cell carbohydrate biosynthesis and growth by catalyzing the interconversion between UDP-glucose and UDP-galactose. This enzyme is involved in channeling UDP-D-galactose into cell wall polymers which is required for the galactosylation of xyloglucan (XyG) and type II arabinogalactan (AGII) [50]. Finally, we propose genes encoding for a polygalacturonase (Zm00001d034727) for the QTL for FA at 1.11 and two genes encoding pectinesterase inhibitors (Zm00001d016738 and Zm00001d016741) for QTL in bin 5.04. These enzymes are involved in physiological processes such as modification of different cell wall polymers and cell wall modulation processes during development and stress responses [51, 52]. In relation to DFAT, we propose five genes as the most probable candidates out of the ones found in the confidence intervals of QTL. We highlight an aldose-1-epimerase gene as probable candidate gene for QTLs for DFA 8–5 l and DFA 8–5 at bin 1.11 because accumulation of the aldose-1-epimerase has been related to cell wall modification, rearrangement and stiffening [53]. For the QTL at bin 3.04, we propose a gene enconding a Gibbellerin 2-oxidase (Zm00001d040737) that irreversibly catalyze the deactivation of bioactive gibbellerin because Wuddineh et al. [54] demonstrated that lignification and biomass recalcitrance could be optimized by targeting gibberellin biosynthesis. We also suggest a candidate gene that encodes an endoplasmic reticulum transporter vesicle protein (Zm00001d043052) for the QTL in bin 3.06. The dynamic structure of the cell wall is controlled by polysaccharide modification, and the pectic and non-cellulosic polysaccharide constituents of plant cell walls are made within endoplasmic reticulum (ER)-Golgi apparatus and exported to the cell surface [55]. Finally, a polygacturonase gene is proposed as candidate gene for the QTL at bin 10.3 because its implication in cell wall loosening during growth and development by the modification and/or breakdown of different polymers [56].

## Conclusions

Overall, new and known genomic regions associated with the accumulation of cell wall-bound hydroxycinnamic acids in the maize pith were revealed, and could have impact on hydroxycinnamate content across different genetic backgrounds. Genes involved in cell wall modulation were proposed as candidate genes for cell wall-bound hydroxycinnamate accumulation. However, we cannot rule out that some others uncharacterized genes linked to significant SNPs could be implicated in dimers formation and arobinoxylan feruloylation.

The effects of individual SNPs significantly associated with hydroxycinnamate content were low, and each SNP explained a low percentage of total genetic variability. Therefore, the most appropriate marker-based breeding strategy to increase hydroxycinnamate content would be the genomic selection, because markers evenly distributed throughout the entire genome would better estimate a breeding value than few markers linked to QTL with a modest contribution to the genetic variability.

## Methods

### Plant materials and experimental design

Most diversity available in the worldwide public breeding sector is represented in the diversity panel, comprising 302 inbred lines, evaluated [57, 58]. A subset of 270 inbred lines from this diversity panel was evaluated for cell wall-bound hydroxycinnamates (mostly due to seeds germinations problems). Field trials using 18 × 15 α-lattice experimental designs with two replicates were performed in Pontevedra, Spain, in 2010 and 2011. Plot characteristics and agronomic practices were as described by Samayoa et al. [23]. The plant material was obtained from the North Central Regional Plant Introduction Station from Iowa State University following the seeds import law applicable for Spain. The list of inbred lines used in this study along with their accession identifications at the North Central Regional Plant Introduction Station (NCRPIS) is available as additional file in Samayoa et al. [59].

### Biochemical determinations

The second internodes below the main ear from five plants per plot were collected 30 days after silking. Samples were frozen at − 20 °C. The pith was manually detached, lyophilized, and ground in a Wiley mill with a 0.75 mm screen. Ground pith samples were maintained at 5 °C until biochemical analyses. Hydroxycinnamates quantification was done following a recently optimized protocol by Santiago et al. [60]. The identities of FA dimers were confirmed by a comparison with the authentic 5–5 standard or published retention times and UV spectra [61]. The total diferulate content (DFAT) was calculated as the sum of the following three identified and quantified DFA isomers: DFA 8–*O*–4, DFA 5–5-, and DFA 8–5. The DFA 8–5 concentration was calculated as the sum of 8–5-cyclic (or benzofuran)-DFA and 8–5-noncyclic (or open). Additionally, MONOTOT represents the sum of all monomers (PCA and FA), while FENTOT refers to the sum of all hydroxycinnamate (monomers and dimers).

### Statistical analysis

Inbred lines were previously genotyped with a unique set of SNP markers derived from the Illumina maize 50 k array and a genopying-by-sequencing (GBS) strategy (90, 91). The two resulting datasets were combined. The SNPs with more than 20% missing data and a minor allele frequency less than 5% were omitted. Heterozygous genotypes were considered missing data. After filtering, 246,477 SNPs distributed across the maize genome were retained [23, 62, 63].

#### Best linear unbiased estimator

Each trial was analyzed separately according to the mixed model procedure (PROC MIXED) of the SAS program (version 9.4) [64] and the best linear unbiased estimators for each inbred line were calculated based on the combined data for the 2-year analysis. Inbred lines were considered as the fixed effect, while years, replicates and blocks within replicates were random effects.

#### Heritabilities and correlations

Heritabilities (ĥ^2^) were estimated for each trait on a family-mean basis as previously described [65]. The genetic (*r*_g_) and phenotypic (*r*_p_) correlation coefficients between traits were calculated using REML estimates according to a published SAS mixed model procedure [66].

#### Association analysis

A genome-wide association analysis was completed with Tassel 5 [67] based on a mixed linear model using a genotype-phenotype matrix and a kinship matrix [26] as a covariate. Among the mixed linear model options, we used the optimum compression level and P3D to estimate the variance component. An established subsampling method [23] was applied to identify SNPs with the most robust associations (RMIP).

The genetic kinship matrix used for this GWAS was previously published [26], and was estimated using a subset of 5000 SNPs (with no missing genotypes) that were distributed almost evenly across the whole maize genome.

#### Candidate gene selection

Following previous studies performed with the same genetic material [23] and the LD decay observed in the regions surrounding significant SNPs, we considered a +/− 150 kbp confidence interval region around each significant SNP. LD between SNPs separated by more than 150 Kbp is inappreciable (*r*^2^ < 0.1) in at least 90% of cases [62]. In case confidence intervals of two SNPs overlapped, they were assigned to a single QTL. The two described genes that delimit the 300 kbp region around the SNP in the reference genome assembly version 2 were positioned in version 4 of the reference genome, and all genes contained in the region delimited by those genes were then identified and characterized based on the maize B73 reference genome assembly (version 4) available on the MaizeGDB browser [25].

## Supplementary information

**Additional file 1:** Means of 270 association-panel inbreds for contents of cell-wall bound hydroxycinnamates evaluated in two years.

**Additional file 2:** Colocalizations between genomic regions associated with cell-wall bound hydroxycinnamates in the current study and published QTLs for cell-wall traits, resistance to pests, forage digestibility and bioethanol production.

## Data Availability

The data sets used and/or analysed during the current study will be available upon reasonable request to the corresponding author. Vegetal materials are distributed to the scientific community by the NCRPIS upon request (https://. www.maizegdb.org/data_center/stock?id=3100329).

## Abbreviations

DFA 5–5: 5–5 diferulic acid
DFA 8–5: sum (DFA8–5 l + DFA8–5b)
DFA 8–5 l: 8–5 linear diferulic acid
DFA 8–5b: 8–5 benzofuran diferulic acid
DFA 8–*O*–4: 8–*O*–4 diferulic acid
DFAT: total diferulates (DFA 5–5 + DFA 8–*O*–4 + DFA 8–5b + DFA 8–5 l)
FA: ferulic acid
FENTOT: total hydroxycinnamates (PCA+ FA+ DFAT)
GWAS: Genome-Wide Association
LD: Linkage disequilibrium
MCB: Mediterranean Corn Borer
MONOTOT: total monomers (PCA + FA)
PCA: *p*-coumaric acid
SNP: Single Nucleotide Polimorfism
